# Suicide attempts in Spain according to prehospital healthcare emergency records

**DOI:** 10.1371/journal.pone.0195370

**Published:** 2018-04-09

**Authors:** Yolanda Mejías-Martín, Celia Martí-García, Candela Rodríguez-Mejías, Juan Pablo Valencia-Quintero, M. Paz García-Caro, Juan de Dios Luna

**Affiliations:** 1 Department of Mental Health, University Hospital Complex of Granada, Granada, Spain; 2 Department of Nursing, Faculty of Health Sciences, University of Malaga, Malaga, Spain; 3 Department of Intensive Care, University Hospital Complex of Granada, Granada, Spain; 4 Department of Nursing, Faculty of Health Sciences, University of Granada, Granada, Spain; 5 Biostatistics Unit, Faculty of Medicine, University of Granada, Granada, Spain; Karolinska Institutet, SWEDEN

## Abstract

**Objective:**

To analyze the number and characteristics of suicide attempts by reviewing records of the public emergency healthcare service information system.

**Method:**

A retrospective observational study was conducted of emergency telephone calls received between January 1 2007 and December 31 2013 throughout the Andalusia region (Southern Spain). Cases were selected based on phone operator or healthcare team labeling. Data were analyzed on the characteristics of the individuals, the timing and severity of attempts, their prioritization, and their outcome.

**Results:**

Between January 1 2007 and December 31 2013, 20.942 calls related to suicide attempts were recorded, a rate of 34.7 attempts per 100,000 inhabitants. Most cases were classified by the public emergency healthcare service (Empresa Pública de Emergencias Sanitarias, EPES) as code X84 (The International Statistical Classification of Diseases, tenth revision, ICD-10) or 305(The International Statistical Classification of Diseases, ninth revision, ICD-9). Attempts were more frequent in the 35-49-year age group and there were similar proportions of males and females. The lowest number of calls for suicide attempts were in 2007 and the highest in 2013. Calls were more frequent during the summer months, at weekends, and between 16:00 and 23:00 h. The likelihood of evacuation to the hospital emergency department was almost two-fold lower in over 65-yr-olds than in younger individuals.

Significant (*ƿ =* 0.001) gender differences were found in call outcome and prioritization. The most influential factor for evacuation to a hospital emergency department was the code assigned by the attending healthcare team.

**Conclusions:**

Information obtained from extra-hospital emergency services provides valuable data on the characteristics and timing of calls related to suicide attempts, complementing information from hospital emergency departments or population surveys. There is a need to standardize the definition and recording of a suicide attempt.

## Introduction

During 2012, suicide represented 50% of violent deaths in males and 71% in females worldwide [1]. Suicide has been the most frequent external cause of death in Spain since 2008, according to the National Statistics Institute [2]. The risk of suicide is considered higher for individuals who have made a previous attempt than for those who have not, regardless of age and sex [3, 4]. The WHO has reported that the prevalence of self-reported suicide attempts is around 4/1,000 adults [1].

Suicide attempts have major personal, family, social, and economic repercussions [1, 5]. Suicide rates are recorded in most developed countries, but there is little information on suicide attempts [1, 6, 7]. The identification of a suicide attempt is complex, hampering data gathering [8], because of difficulties in establishing its true intent [9]. The WHO proposed the term “non-deadly suicide behavior” for suicidal actions that do not end in death [8]. The International Statistical Classification of Diseases, tenth revision (ICD-10) labels voluntary poisoning and self-harm behaviors with the codes X60-X84.

The dimension and characteristics of suicide attempts have been assessed by general population surveys in Europe [10, 11]and Spain[12, 13] and by the analysis of data obtained from hospital emergency department records [14, 15]. However, there are known to be more suicide attempts than those leading to hospital emergency department admission [16, 17], and around half of individuals visiting the emergency department after self-harm are not hospitalized and are not taken into account in hospital-based surveys of suicide attempts [18]. It is therefore possible to obtain further and more complete information may be obtained by studying the records of prehospital emergency services. In Spain, the prehospital medical emergence system forms part of the national public health service but is managed and organized by each Autonomous Community in the country, and there is no single national database. In Andalusia, the Spanish Autonomous Community with the largest population (more than 8 million inhabitants during the study period), prehospital emergency medical services are provided by the public emergency healthcare service (Empresa Pública de Emergencias Sanitarias [EPES])[19]. EPES coordination centers (one in each province of Andalusia), receive and manage emergency telephone calls and order the most appropriate mobile resource, assigning a priority level to each call [20, 21]. Consequently, all data on calls and on the activity of responding emergency teams in Andalusia are kept in a single database.

The objective of this study was to analyze the number and characteristics of suicide attempts between 2007 and 2013 by reviewing records of the public emergency healthcare service information system.

## Methods

An observational, cross-sectional, and retrospective study was conducted of suicide attempts recorded in the EPES of Andalusia between January 1 2007 and December 31 2013.

### Sources of information and selection of records

The same number (061 or 112) is used by the coordinating center in each of the eight provinces of Andalusia and is attended 24 h/day, being available to all members of the public, other emergency services, and law enforcement organizations [22]. Calls can be received from an informant or from the person making the suicide attempt. Calls are managed by the medical phone operator and coordinator, and the responses to callers range from health advice to the immediate activation of mobile resources according to the priority assigned to the call. Prioritization criteria are based on the severity of the medical situation and appropriate response time. Data were obtained from the EPES Information System (SIEPES) on calls labeled by the phone operators and/or the emergency healthcare teams as suicide attempts, defined according to a common protocol (see below).

#### Suicide attempt definition

The SIEPES protocol establishes the following criteria:

- Calls in which the word “suicide” (or analogous term) is mentioned during responses to the standard questions asked by the phone operator are automatically labeled code X84 [16, 22], considered to represent labels from X60 to X84 of the ICD-10.
- After attending the emergency, the extra-hospital healthcare team labels the case as a suicide attempt or assigns an ICD-9 code for the attempt mechanism. i.e., 305.4, 305.8, E950 -E959, or E980 -E989 (Table 1).

**Table 1:** ICD 9 and ICD 10 codes that define a case as “suicide attempt”.

**ICD10**	**X84**	**Intentional self-harm by unspecified means**
**ICD 9**	305 (305.4, 305.8)	Nondependent abuse of drugs. (Nondependent sedative, hypnotic or anxiolytic abuse. Nondependent antidepressant-type abuse)
	E950—E959	Suicide and self-inflicted injury
	E980—E989	Injury undetermined whether accidentally or purposely inflicted
	969(969.0, .1, .2, .3, .4, .5, .8, .9)	Poisoning by psychotropic agents
	300.9	Unspecified non-psychotic mental disorder (Suicidal Tendencies)
	V62.84	Suicidal ideations

In the present study, data were also gathered on codes 969, 300.9, and V62.84, but only when these were associated with X84 or the aforementioned ICD9 codes in the database (Table 1). These codes were included because they do not rule out a suicide attempt, given that healthcare professionals may label the clinical event but not identify and document a suicide attempt when the intentionality is unclear [7,14, 16].

### Study variables

1. Sex, age, Andalusian province (Almeria, Cadiz, Cordoba, Huelva, Granada, Jaen, Malaga, and Seville), timing (year, month, day, and time). Rates were based on a total accumulated population size for the Andalusia region of 58249966 over the seven-year period, ranging between 8059461 and 8449985 for each year under study (see S1 Table). Population data from the Continuous Census of the National Statistics Institute were used to calculate the rates for each year.
2. Suicide attempt call outcomes and prioritization. In each coordinating center, calls were classified by grouping the codes assigned (n = 67) as follows: no action taken by healthcare team, cancellation of resource, evacuation to hospital, refusal of care, death, *in situ* resolution, or referral to another professional. The level of prioritization was classified as: emergency (threat to life or vital function of the patient if immediate effective action is not taken), undelayable emergency (action must be taken as quickly as possible, but there is no threat to life), delayable emergency, or non-urgent emergency.

### Statistical analysis

A descriptive analysis was performed, calculating percentages for qualitative variables and means with standard deviation (SD) for quantitative data. Suicide attempt rates were computed by sex, age, province, and year, and the Poisson homogeneity test was performed, comparing the temporal distribution of attempts with a theoretical uniform distribution. Associations among qualitative variables were analyzed using contingency tables and the χ^2^ test. One-way ANOVA was used to compare mean values of qualitative and quantitative variables, performing pair wise comparisons when the result of the global test was significant and there were more than two groups.

Binary logistic regression was used to analyze the association of evacuation to hospital with each study variable individually (non-adjusted model) and in combination (adjusted model), yielding odds ratios, 95% confidence intervals, and *p* values. This analysis was conducted after excluding emergencies in which death occurred *in situ*, because there was no evacuation in these cases (n = 516). A sensitivity study to evaluate effects of the possible overrepresentation of Malaga was performed by repeating all association analyses with and without data for this province, finding no significant difference between them. P < 0.05 was considered significant. Stata 14.1 was used for statistical analyses [23].

### Ethical aspects

The content of the database used was anonymized to guarantee compliance with Spanish personal data protection legislation (Law 15/1999). The database was only accessed by the study researchers. The project was approved by the Granada University Health Complex and EPES research ethics committees.

## Results

In the region of Andalusia, out of 6,608,031 calls received by the EPES between January 12007 and December 312013, 20,942(0.31%) were identified as suicide attempts in this study, a rate of 36 attempts per 100,000 inhabitants. Code X84 was assigned to 76.31% of cases and code 305to 16.57% (Table 2).

**Table 2:** Frequency of ICD 9 and ICD 10 codes that define a case as “suicide attempt”.

CODES	DESCRIPTION	n (%)
**ICD10X84**	**Intentional self-harm by unspecified means**	**15,980(76.31)**
**ICD 9 305**	**Nondependent abuse of drugs**	**3,470 (16.57)**
305.4	Nondependent sedative, hypnotic or anxiolytic abuse	3,144 (90.61)
305.8	Nondependent antidepressant-type abuse	396 (11.41)
**ICD9E950—E959**	**Suicide and self-inflicted injury**	**1,331 (6.36)**
E950	Suicide and self-inflicted poisoning by solid or liquid substances `	411 (30.88)
E951	Suicide and self-inflicted poisoning by gases in domestic use	16 (1.2)
E952	Suicide and self-inflicted poisoning by other gases and vapors	12 (0.9)
E953	Suicide and self-inflicted injury by hanging, strangulation, and suffocation	198 (14.88)
E954	Suicide and self-inflicted injury by submersion (drowning)	4 (0.3)
E955	Suicide and self-inflicted injury by firearms, air guns and explosives	26 (1.95)
E956	Suicide and self-inflicted injury by cutting and piercing instrument	179 (13.45)
E957	Suicide and self-inflicted injury by jumping from high places	56 (4.21)
E958	Suicide and self-inflicted injury by other and unspecified means	273 (20.51)
E959	Late effects of self-inflicted injury	167 (12.55)
**ICD9 E980—E989**	**Injury undetermined whether accidentally or purposely inflicted**	**709 (3.39)**
E980	Poisoning by solid or liquid substances undetermined whether accidentally or purposely inflicted	82 (11.57)
E981	Poisoning by gases in domestic use undetermined whether accidentally or purposely inflicted	9 (1.27)
E982	Poisoning by other gases undetermined whether accidentally or purposely inflicted	16 (2.26)
E983	Hanging, strangulation or suffocation undetermined whether accidentally or purposely inflicted	229(32.3)
E984	Submersion (drowning), undetermined whether accidentally or purposely inflicted	16 (2.26)
E985	Injury by firearms air guns and explosives undetermined whether accidentally or purposely inflicted	34 (4.8)
E986	Injury by cutting and piercing instruments, undetermined whether accidentally or purposely inflicted	58 (8.18)
E987	Falling from high place undetermined whether accidentally or purposely inflicted	142 (20.03)
E988	Injury by other and unspecified means undetermined whether accidentally or purposely inflicted	17 (2.4)
E989	Late effects of injury, undetermined whether accidentally or purposely inflicted	110 (15.51)
**ICD9 969**	**Poisoning by psychotropic agents**	**1,443 (6.89)**
969.0	Poisoning by antidepressants	183 (12.69)
969.1	Poisoning by phenothiazine-based tranquilizers	5 (0.35)
969.2	Poisoning by butyrophenone-based tranquilizers	3 (0.21)
969.3	Poisoning by other antipsychotics neuroleptics and major tranquilizers	20 (1.39)
969.4	Poisoning by benzodiazepine-based tranquilizers	1,250 (86.69)
969.5	Poisoning by other tranquilizers	5 (0.35)
969.8	Poisoning by other specified psychotropic agents	6 (0.42)
969.9	Poisoning by unspecified psychotropic agent	33 (2.29)
**ICD9300.9**	**Unspecified non-psychotic mental disorder (Suicidal Tendencies)**	**1,045 (4.99)**
**ICD9V62.84**	**Suicidal ideations**	**67 (0.32)**

### Characteristics of recorded suicide attempts

The mean (SD) age of the individuals attempting suicide was 42.72 (±15.94) yrs. The rate increased with higher age up to 45 yrs and then decreased, being lowest among over 80-yr-olds. Among younger age groups, a peak rate of 6.60% was observed in those aged 20–24 years in comparison to 0.04% in those aged 15–19 years. Just over half of suicide attempt-related calls in the region were for females (50.98%), with a rate of 36.3 per 100,000 inhabitants versus 33.1 per 100,000 inhabitants for males, a ratio of 1.10: 1. Among provinces, the female: male ratio was highest in Malaga (1.23:1), which also had highest age-adjusted suicide attempt rate (68.07 per 100,000 inhabitants), much higher than the province with the lowest rate (Huelva: 17.60 per 100,000 inhabitants) (Table 3 and S2 Table).

**Table 3:** Description of calls related to suicide attempts in Andalusia between 2007 and 2013.

		n (%)	Rates^a^			n (%)	Rates^a^
**Sex**	Male	9,544(45.57)	33.1***	**Year**	2007	2,612(12.47)	32.4***
	Female	10,676(50.98)	36.3		2008	3,034(14.49)	37.0
	Unknown	722(3.45)			2009	3,012(14.38)	36.3
					2010	2,867(13.69)	34.2
					2011	2,953(14.10)	35.1
					2012	3,051(14.57)	36.1
					2013	3,413(16.30)	40.4
**Age**	15–19	772(0.04)	6.0***	**Month**	January	1,507(7.20)***	
	20–24	1,261(6.60)	33.8		February	1,575(7.52)	
	25–29	1,685(8.82)	38.4		March	1,866(8.91)	
	30–34	2,200(11.51)	44.6		April	1,669(7.97)	
	35–39	2,533(13.26)	51.8		May	1,802(8.60)	
	40–44	2,735(14.31)	57.6		June	1,845(8.81)	
	45–49	2,260(11.83)	51.9		July	1,936(9.24)	
	50–54	1,817(9.51)	49.0		August	1,985(9.48)	
	55–59	1,032(5.40)	33.4		September	1,767(8.44)	
	60–64	711(3.72)	25.2		October	1,703(8.13)	
	65–69	562(2.94)	23.2		November	1,574(7.52)	
	70–74	457(2.39)	21.4		December	1,713(8.18)	
	75–79	385(2.01)	19.9				
	80–84	403(2.11)	30.7				
	≥85	296(1.55)	30.9				
	Unknown	1,833(8.75)					
**Province**^b^	Almería	1,531(7.31)	31.0***	**Day**	Monday	3,033(14.77)***	
	Cádiz	2,899(13.84)	31.1		Tuesday	2,877(13.74)	
	Córdoba	1,564(7.47)	25.0		Wednesday	2,862(13.67)	
	Granada	2,080(9.93)	32.0		Thursday	2,874(13.72)	
	Huelva	779(3.72)	15.3		Friday	2,866(13.69)	
	Jaén	1,046(4.99)	21.2		Saturday	3,153(15.06)	
	Malaga	7,029(33.56)	60.0		Sunday	3,217(15.36)	
	Seville	4,014(19.17)	24.2				
**Call outcome**	No action of the healthcare team	875(4.18)		**Time slot**	0–7	3,632(17.35)***	
	Resource cancellation	250(1.19)			8–15	7,532(35.98)	
	Evacuation	15,156(72.37)			16–23	9,768(46.66)	
	Denial to be attended	409(1.96)		**Priority**	Emergency	2,891(13.81)	
	Death	516(2.46)			Undelayable emergency	17,606(84.07)	
	In situ resolution	2,728(13.05)			Delayable emergency	361(1.72)	
	Referral to another professional	966(4.61)			Not urgent	82(0.39)	
	Unknown	42(0.20)			Unknown	2(0.00)	

*** Global test forcount and rates comparison P<0.001.

^a:^ Number of suicide attempts per 100,000 inhabitants.

^b:^ Rates per province standardized by age and sex.

**Fig 1:**
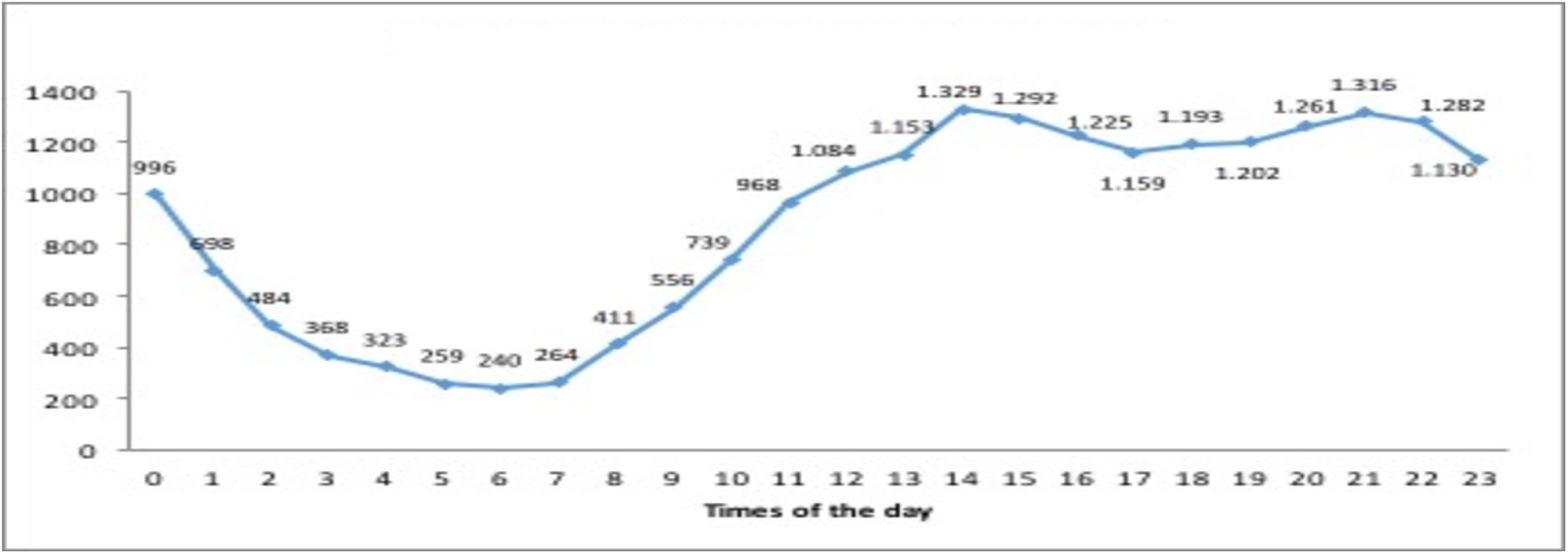
Number of suicide attempts by time of day.

The lowest call rate for suicide attempts was in 2007 (32.4 per 100,000 inhabitants) and the highest in 2013 (40.4 per 100,000 inhabitants), although there was no linear increase over the study period. As shown in Table 3, suicide attempt calls were more frequent during the summer months (9.24% in July and 9.48% in August), at weekends (15.06% on Saturday and 15.36% on Sunday), and between 16:00–23:00 (46.66%). The call rate was highest at 14:00 (6.35%) (Fig 1) and remained elevated until midnight, more markedly between 16:00 and 23:00, followed by a marked reduction until 8:00 h.

Out of the 20,942 cases of attempted suicide recorded, 84.07% were classified as non-delayable. The patient was evacuated or taken to hospital in 72.52% of cases, while the patient died (before or after treatment by the mobile team) in 2.47% of cases (Table 3).

### Characteristics of suicide attempts by sex

Attempts were analyzed by sex (S2 Table), finding significant (*ƿ =* 0.001) differences in call outcome and prioritization. Females were more frequently evacuated to a hospital center (74.53%), and the call was more frequently resolved *in situ* (13.40%) for females than for males, while the outcome was death for 4% of males *versus* 0.98% of females, and the call was prioritized as “emergency” for 15.03% of males *versus* 12.94% of females.

### Analysis of hospital admissions

Table 4 summarizes data on evacuation to a hospital emergency department as a function of selected factors; a full list is exhibited in S3 Table.

**Table 4:** Logistic regression of suicide attempts with respect to evacuation (summarized).

		Non-adjusted model	Adjusted model
		OR (95% CI)	OR (95% CI)
Province	**Almeria**	Reference	Reference
	**Remaining Provinces**	0.49 (0.42;0.57)***	0.54 (0.46;0.63)***
Age	**<65**	Reference	Reference
	**> = 65**	0.63 (0.57;0.69)***	0.53 (0.47;0.59)***
Sex	**Male**	Reference	Reference
	**Female**	1.05 (0.98;1.12)	1.05 (0.97;1.12)
Time of Call	**0**	Reference	Reference
	**1**	1.09 (0.88;1.35)	1.08 (0.85;1.37)
	**2**	1.15 (0.91;1.47)	1.19 (0.91;1.55)
	**3**	1.45 (1.1;1.92)**	1.90 (1.37;2.64)***
	**4**	1.22 (0.92;1.62)	1.32 (0.96;1.8)
	**5**	1.14 (0.84;1.55)	1.06 (0.76;1.48)
	**6**	0.91 (0.67;1.23)	0.92 (0.65;1.31)
	**7**	1.42 (1.03;1.96)*	1.20 (0.84;1.73)
	**8**	2.29 (1.7;3.1)***	2.37 (1.69;3.34)***
	**9**	1.46 (1.15;1.87)**	1.50 (1.14;1.99)**
	**10**	1.56 (1.25;1.95)***	1.48 (1.15;1.9)**
	**11**	1.5 (1.22;1.84)***	1.48 (1.17;1.86)**
	**12**	1.46 (1.2;1.78)***	1.41 (1.12;1.76)**
	**13**	1.38 (1.14;1.67)**	1.38 (1.11;1.72)**
	**14**	1.44 (1.2;1.74)***	1.34 (1.09;1.66)**
	**15**	1.33 (1.1;1.6)**	1.36 (1.1;1.68)**
	**16**	1.35 (1.12;1.63)**	1.31 (1.06;1.62)*
	**17**	1.35 (1.11;1.63)**	1.33 (1.07;1.64)*
	**18**	1.34 (1.1;1.61)**	1.29 (1.04;1.59)*
	**19**	1.36 (1.12;1.64)**	1.31 (1.06;1.62)*
	**20**	1.26 (1.05;1.52)*	1.24 (1.01;1.53)*
	**21**	1.14 (0.95;1.37)	1.15 (0.94;1.42)
	**22**	1.2 (0.99;1.44)	1.17 (0.95;1.44)
	**23**	1.1 (0.91;1.33)	1.13 (0.91;1.39)
ICD-10:X84	**No**	Reference	Reference
	**Yes**	0.30(0.28;0.33)***	1.26 (1.03;1.55)*
ICD-9:950	**No**	Reference	Reference
	**Yes**	6.61 (5.11;8.53)***	7.89 (5.93;10.49)***
ICD-9:980	**No**	Reference	Reference
	**Yes**	2.63 (2.06;3.36)***	3.56 (2.63;4.82)***
ICD-9:305	**No**	Reference	Reference
	**Yes**	2.43 (2.19;2.69)***	3.06 (2.5;3.75)***
ICD-9:969	**No**	Reference	Reference
	**Yes**	6.90 (5.45;8.74)***	8.45 (6.49;11.01)***
ICD9:300.9	**No**	Reference	Reference
	**Yes**	6.19 (4.74;8.08)***	7.79 (5.89;10.3)***
ICD9:V62.84	**No**	Reference	Reference
	**Yes**	7.39 (2.32;23.52)**	9.89 (3.08;31.77)***
Priority	**Not Maximum**	Reference	Reference
	**Maximum**	2.93 (2.6;3.31)***	1.58 (1.35;1.84)***

*p˂0.05.

**p˂0.01.

***p˂0.001.

There was a two-fold lower likelihood of evacuation in over-65-yr-olds than in younger patients. Evacuation to hospital was most frequently ordered at 3:00 and 8:00 in the morning, followed by a decrease until midnight. The most influential factor was the code assigned by the attending healthcare team; the likelihood of hospital evacuation was at least 2.5 fold higher when any of the following codes were assigned (in descending order of influence): 950, 969, 300.9, V62.84, 980, or 305. No differences were found in evacuation to hospital as a function of sex, day of the week, month or year.

## Discussion

In this seven-year study of prehospital emergency services in Andalusia, the highest estimated suicide attempt rate among adults in our region was in the most recent year studied (2013), indicating that there are four suicide attempts for every actual suicide. In contrast, the WHO reported in 2014 that there are at least 20 suicide attempts for every actual suicide [1]. Suicide attempts represent a small percentage of emergency calls but affect a young population and there are few specific protocols for the emergency response to this situation [1, 24].

The present study focused exclusively on pre-hospital emergency services using information retrieved from the EPES database. Most published studies on suicide attempts in the healthcare setting are based on data from hospital emergency departments, and we could trace no published research that used information from pre-hospital emergency services in other countries, hampering comparison of our results with findings elsewhere.

In general, the suicide attempt rate (per 100,000 inhabitants) was lower in the present population (mean of 36.0, range, 32.4–40.4 in different years) than in other parts of Spain [14, 25–27], other European countries [28, 29] and the USA (163.1–173.8 per year)[30].

Virtually no gender difference was observed in the present study, with the likelihood of a suicide attempt being only 1.05-fold higher for females. However, a higher frequency of suicide attempts among females than males has been observed in other Spanish regions and European countries [14, 22, 25–29] and in the USA, where the American Association of Suicidology [31] reported three female attempts for each male attempt between 2008 and 2015. These discrepancies might be attributable to cultural differences, although this proposition is not supported by the variability in the reported gender balance of attempts among different populations within Spain [14, 25–27]. It should first be explored whether variations are attributable to differences in methodology and/or the type and quality of data sources/data.

The mean age of adults attempting suicide was higher in Andalusia than has been reported in other Spanish regions (Madrid, Galicia, and Murcia) [14, 25–27]and in some other European countries [29]and the USA [30]. Results in other Spanish and European populations are closer to those obtained in the USA in comparison to the present findings, but the variability among studies is very high. In Andalusia, a peak in recorded suicide attempts was observed between 20–24 years of age, older than the ages at which a peak has been observed in other regions or countries. Thus, Canner et al [30] reported that visits to the emergency department for suicide attempt or self-harm peaked between the ages of 15 and 19 years in the USA. Various factors may help to explain this discrepancy, including our utilization of pre-hospital medical service *versus* emergency department records, and it has also been proposed that the classification of acts as intentional may vary among healthcare professionals, especially in relation to younger individuals [32, 33]. However, further research is required to elucidate the reasons for this difference in age profile.

The number of calls for suicide attempts was higher in Malaga than in the other provinces, and further investigation is warranted in this province to examine possible explanations for this finding.

The most influential factor for evacuation to hospital was the code assigned by the attending emergency team according to the severity of the suicide attempt. The likelihood of evacuation was lesser among over 65-year-olds, suggesting that protocols for evacuation to hospital may require revision, given evidence of a higher post-attempt mortality rate in older individuals [26].

In the present study, suicide attempts were more frequent during the very hot summer months, and a review by Deisenhammer [34] proposed that attempts were more frequent on days with higher temperatures and more hours of sun; however, the findings were not conclusive, and some of the reported data were contradictory. In our Southern Spanish population, attempts were more frequent on public holidays and at weekends, as also observed in a region of Northern Spain [35]. Studies in different parts of Spain have reported that suicide attempts treated by hospital emergency departments are more frequent at weekends and are often associated with the consumption of recreational drugs and/or alcohol [26, 35, 36]. An association between alcohol use and suicide attempts is well documented [37–39], and alcohol consumption is especially high at weekends in Spain, including increasingly popular binge-drinking gatherings of adolescents and young adults [40,41]. However, the present data do not allow conclusions to be drawn on a possible causal relationship between the increased consumption of alcohol and the higher frequency of suicide attempts observed at weekends.

Finally, the procedure followed to meet our study objective revealed problems in identifying suicide attempts from prehospital emergency service records, which do not appear to pay special attention to this issue. This may result in an underestimation of suicide attempt rates, as also acknowledged by Clements et al [17]. Steps were taken to reduce the loss of cases in the present study by including those labeled as a suicide attempt by the call operator/coordinator as well as by the emergency healthcare team *in situ*, which may be less likely to identify a case not originally assigned the X84 code given their focus on the severity and treatment of injury rather than its cause or intentionality.

There are few published data on prehospital emergency calls for a suicide attempt. In their analysis of psychiatric prehospital emergencies, Panjonk et al [42] found that 26% were for suicide attempt or suicidal ideation.

Although the evidence on suicidal behavior appears generally inadequate, it suggests the need to develop specific healthcare strategies, assigning particular importance to the extra-hospital emergency response.

### Limitations

Potential study limitations include the possibility of selection and/or information bias due to its retrospective and cross-sectional design. Although a highly standardized and controlled database was utilized, numerous professionals were involved in its generation and transcription, so that the influence of non-controlled variables cannot be ruled out. Furthermore, our results should be interpreted with caution, because only one database was used and there was no follow-up study of callers attended by the emergency team. In addition, it was not possible to identify multiple contacts by the same individual, given the anonymous nature of the database. Finally, account should be taken of cases lost when no advance call is made to the emergency service and individuals reach the hospital emergency department by other means or use a private network (highly infrequent in our setting), or when the suicide method involves a traffic accident or fall, among other mechanisms that may mask the intention of the victim.

## Conclusion

Information obtained from extra-hospital emergency services provides valuable data on the characteristics and timing of suicide attempts and on the priority assigned to cases and their outcome. These data complement information gathered on suicide attempts in studies of hospital emergency departments or in population surveys.

There is a need for research to establish a consensus on the definition and recording of suicide attempts in order to advance knowledge on the scale and characteristics of this phenomenon.

## Supporting information

S1 Table Suicide attempt rates by study variable with reference populations. (DOCX)

S2 Table Timing of suicide attempts by sex. (DOCX)

S3 Table Logistic regression of suicide attempt with respect to evacuation (complete). (DOCX)
