# Supplementary material for: Suicide attempts in Spain according to prehospital healthcare emergency records
Source: PLoS One. 2018 Apr 9;13(4):e0195370. doi: 10.1371/journal.pone.0195370 (PMC5891009; doi:10.1371/journal.pone.0195370)
Supplement: S1 Table — (DOCX) [file pone.0195370.s001.docx]

| **S1 Table** **Suicide attempt rates by study variable with reference populations** | | | | | | | | | | | | | |
| --- | --- | --- | --- | --- | --- | --- | --- | --- | --- | --- | --- | --- | --- |
|  | | **Suicide Attempts (n)** | **Population** | **Rate** |  | | | **Suicide Attempts (n)** | | **Population** | | **Rate** | |
| **Sex** | Male | 9,544 | 28849555 | 33.1 | **Year** | 2007 | | 2,612 | | 8059461 | | 32.4 | |
|  | Female | 10,676 | 29400411 | 36.3 |  | 2008 | | 3,034 | | 8202220 | | 37.0 | |
|  | Total | 20,220 | 58249966 | 34.7 |  | 2009 | | 3,012 | | 8302923 | | 36.3 | |
|  |  |  |  |  |  | 2010 | | 2,867 | | 8370975 | | 34.2 | |
|  |  |  |  |  |  | 2011 | | 2,953 | | 8424102 | | 35.1 | |
|  |  |  |  |  |  | 2012 | | 3,051 | | 8449985 | | 36.1 | |
|  |  |  |  |  |  | 2013 | | 3,413 | | 8440300 | | 40.4 | |
|  |  |  |  |  |  | Total | | 20,942 | | 58249966 | | 36.0 | |
| **Age** | 15-19 | 772 | 12830012 | 6.0 | **Province** | Almería | | 1,531 | | 4800621 | | 31.9 | |
|  | 20-24 | 1,261 | 3735260 | 33.8 |  | Cádiz | | 2,899 | | 8622318 | | 33.6 | |
|  | 25-29 | 1,685 | 4384498 | 38.4 |  | Córdoba | | 1,564 | | 5612887 | | 27.9 | |
|  | 30-34 | 2,200 | 4929007 | 44.6 |  | Granada | | 2,080 | | 6377616 | | 32.6 | |
|  | 35-39 | 2,533 | 4888339 | 51.8 |  | Huelva | | 779 | | 3602568 | | 21.6 | |
|  | 40-44 | 2,735 | 4750014 | 57.6 |  | Jaén | | 1,046 | | 4678481 | | 22.4 | |
|  | 45-49 | 2,260 | 4354735 | 51.9 |  | Málaga | | 7,029 | | 11203333 | | 62.7 | |
|  | 50-54 | 1,817 | 3708157 | 49.0 |  | Sevilla | | 4,014 | | 13352142 | | 30.1 | |
|  | 55-59 | 1,032 | 3093204 | 33.4 |  | Total | | 20,942 | | 58249966 | | 36.0 | |
|  | 60-64 | 711 | 2820474 | 25.2 |  |  | |  | |  | |  | |
|  | 65-69 | 562 | 2417834 | 23.2 |  |  | |  | |  | |  | |
|  | 70-74 | 457 | 2136199 | 21.4 |  |  | |  | |  | |  | |
|  | 75-79 | 385 | 1931978 | 19.9 |  |  | |  | |  | |  | |
|  | 80-84 | 403 | 1311087 | 30.7 |  |  | |  | |  | |  | |
|  | ≥85 | 296 | 959168 | 30.9 |  |  | |  | |  | |  | |
|  | Total | 19,109 | 58249966 | 32.8 |  |  | |  | |  | |  | |
|  | | **Suicide Attempts (n)** | | **Population Size** | | **Rates** | | | | Ratio Female/Male | | | |
|  |  | Male | Female | Male | Female | Male | | Female | |  | | | |
| **Province** | Almería | 780 | 741 | 2463844 | 2336777 | 31.7 | | 31.7 | | 1.00 | | | |
|  | Cádiz | 1,401 | 1,354 | 4278586 | 4343732 | 32.7 | | 31.2 | | 0.95 | | | |
|  | Córdoba | 683 | 749 | 2757973 | 2854914 | 24.8 | | 26.2 | | 1.06 | | | |
|  | Granada | 956 | 1,106 | 3154550 | 3223066 | 30.3 | | 34.3 | | 1.13 | | | |
|  | Huelva | 374 | 368 | 1790524 | 1812044 | 20.9 | | 20.3 | | 0.97 | | | |
|  | Jaén | 535 | 487 | 2326643 | 2351838 | 23.0 | | 20.7 | | 0.90 | | | |
|  | Málaga | 3,050 | 3,852 | 5527193 | 5676140 | 55.2 | | 67.9 | | 1.23 | | | |
|  | Sevilla | 1,765 | 2,019 | 6550242 | 6801900 | 26.9 | | 29.7 | | 1.10 | | | |


*p˂0.05**p˂0.01***p˂0.001
