# Supplementary material for: Suicide attempts in Spain according to prehospital healthcare emergency records
Source: PLoS One. 2018 Apr 9;13(4):e0195370. doi: 10.1371/journal.pone.0195370 (PMC5891009; doi:10.1371/journal.pone.0195370)
Supplement: S2 Table — (DOCX) [file pone.0195370.s002.docx]

| **S2 Table. Timing of suicide attempts by sex** | | | | | | |
| --- | --- | --- | --- | --- | --- | --- |
|  | | **Male** | | **Female** | |  |
|  |  | **n** | **(%)** | **n** | **(%)** | ***p*** |
| **Year** | 2007 | 1,245 | (13.04) | 1,281 | (12.00) | 0.087 |
|  | 2008 | 1,402 | (14.69) | 1,542 | (14.44) |  |
|  | 2009 | 1,339 | (14.03) | 1,565 | (14.66) |  |
|  | 2010 | 1,341 | (14.05) | 1,431 | (13.40) |  |
|  | 2011 | 1,350 | (14.15) | 1,514 | (14.18) |  |
|  | 2012 | 1,371 | (14.37) | 1,560 | (14.61) |  |
|  | 2013 | 1,496 | (15.67) | 1,781 | (16.70) |  |
| **Month** | January | 711 | (7.45) | 744 | (6.97) | 0.755 |
|  | February | 714 | (7.48) | 797 | (7.47) |  |
|  | March | 876 | (9.18) | 928 | (8.69) |  |
|  | April | 780 | (8.17) | 834 | (7.81) |  |
|  | May | 831 | (8.71) | 932 | (8.73) |  |
|  | June | 819 | (8.58) | 966 | (9.05) |  |
|  | July | 876 | (9.18) | 998 | (9.35) |  |
|  | August | 903 | (9.46) | 1,014 | (9.50) |  |
|  | September | 808 | (8.47) | 881 | (8.25) |  |
|  | October | 768 | (8.05) | 875 | (8.20) |  |
|  | November | 704 | (7.38) | 808 | (7.57) |  |
|  | December | 754 | (7.90) | 899 | (8.42) |  |
| **Day** | Monday | 1,388 | (14.54) | 1,578 | (14.78) | 0.001 |
|  | Tuesday | 1,356 | (14.21) | 1,430 | (13.39) |  |
|  | Wednesday | 1297 | (13.59) | 1,460 | (13.68) |  |
|  | Thursday | 1,363 | (14.28) | 1,420 | (13.30) |  |
|  | Friday | 1,363 | (14.28) | 1,412 | (13.23) |  |
|  | Saturday | 1,405 | (14.72) | 1,640 | (15.36) |  |
|  | Sunday | 1,372 | (14.38) | 1,736 | (16.26) |  |
| **Province** | Almeria | 780 | (51.28) | 741 | (48.72) | 0.000 |
|  | Cadiz | 1,401 | (50.85) | 1,364 | (49.15) |  |
|  | Cordoba | 683 | (47.70) | 749 | (52.30) |  |
|  | Granada | 956 | (46.36) | 1,106 | (53.64) |  |
|  | Huelva | 374 | (50.40) | 368 | (49.60) |  |
|  | Jaen | 535 | (52.35) | 487 | (47.65) |  |
|  | Malaga | 3,050 | (44.19) | 3,852 | (55.81) |  |
|  | Sevilla | 1,765 | (46.64) | 2,019 | (53.36) |  |
| **Call outcome** | No action of healthcare team | 395 | (4.15) | 437 | (4.10) | 0.000 |
|  | Resource cancellation | 95 | (1.00) | 137 | (1.29) |  |
|  | Evacuation | 6,800 | (71.43) | 7,946 | (74.53) |  |
|  | Refusal of care | 209 | (2.20) | 190 | (1.78) |  |
|  | Death | 381 | (4.00) | 105 | (0.98) |  |
|  | In situ resolution | 1,187 | (12.47) | 1,429 | (13.40) |  |
|  | Referral to another professional | 453 | (4.76) | 417 | (3.91) |  |
| **Priority** | Emergency | 1,343 | (15.03) | 1,381 | (12.94) | 0.000 |
|  | Undelayable emergency | 7,888 | (82.65) | 9,084 | (85.10) |  |
|  | Delayable emergency | 174 | (1.82) | 177 | (1.66) |  |
|  | Non-urgent | 47 | (0.49) | 33 | (0.31) |  |
