# Supplementary material for: Suicide attempts in Spain according to prehospital healthcare emergency records
Source: PLoS One. 2018 Apr 9;13(4):e0195370. doi: 10.1371/journal.pone.0195370 (PMC5891009; doi:10.1371/journal.pone.0195370)
Supplement: S3 Table — (DOCX) [file pone.0195370.s003.docx]

| S3 Table Logistic regression of suicide attempt with respect to evacuation (Complete) | | | |
| --- | --- | --- | --- |
|  | | **Non-adjusted model** | **Adjusted model** |
|  |  | **OR (95% CI)** | **OR (95% CI)** |
| Province | **Almeria** | Reference | Reference |
|  | **Cadiz** | 0.46 (0.39;0.54)*** | 0.53 (0.44;0.64)*** |
|  | **Cordoba** | 0.6 (0.5;0.72)*** | 0.65 (0.52;0.8)*** |
|  | **Granada** | 0.45 (0.38;0.53)*** | 0.52 (0.44;0.63)*** |
|  | **Huelva** | 0.59 (0.47;0.74)*** | 0.53 (0.4;0.7)*** |
|  | **Jaen** | 0.31 (0.25;0.37)*** | 0.39 (0.31;0.48)*** |
|  | **Malaga** | 0.56 (0.48;0.65)*** | 0.57 (0.48;0.67)*** |
|  | **Seville** | 0.45 (0.38;0.53)*** | 0.51 (0.43;0.61)*** |
| Age | **15-19** | Reference | Reference |
|  | **20-24** | 1.17 (0.95;1.44) | 1.15 (0.92;1.44) |
|  | **25-29** | 1 (0.82;1.22) | 0.96 (0.78;1.18) |
|  | **30-39** | 1.19 (0.99;1.45) | 1.16 (0.95;1.42) |
|  | **30-39** | 1.26 (1.04;1.52)* | 1.24 (1.02;1.52)* |
|  | **40-44** | 1.17 (0.97;1.41) | 1.14 (0.94;1.39) |
|  | **45-49** | 1.11 (0.92;1.34) | 1.04 (0.85;1.28) |
|  | **50-54** | 0.93 (0.77;1.13) | 0.86 (0.7;1.05) |
|  | **55-59** | 1.12 (0.9;1.39) | 0.98 (0.78;1.24) |
|  | **60-64** | 1.08 (0.85;1.38) | 0.99 (0.77;1.28) |
|  | **65-69** | 0.79 (0.62;1.01) | 0.63 (0.49;0.82)** |
|  | **70-74** | 0.86 (0.66;1.12) | 0.72 (0.54;0.95)* |
|  | **75-79** | 0.79 (0.6;1.04) | 0.65 (0.48;0.87)** |
|  | **80-84** | 0.69 (0.53;0.91)** | 0.54 (0.4;0.73)*** |
|  | **>=85** | 0.37 (0.28;0.49)*** | 0.26 (0.19;0.36)*** |
| Sex | **Male** | Reference | Reference |
|  | **Female** | 1.05 (0.98;1.12) | 1.05 (0.98;1.13) |
| Year | **2007** | Reference | Reference |
|  | **2008** | 1.09 (0.97;1.24) | 1.12 (0.97;1.29) |
|  | **2009** | 0.97 (0.86;1.1) | 0.98 (0.86;1.13) |
|  | **2010** | 0.93 (0.82;1.05) | 0.97 (0.84;1.12) |
|  | **2011** | 0.93 (0.83;1.05) | 1.02 (0.89;1.17) |
|  | **2012** | 0.86 (0.76;0.97)* | 0.95 (0.83;1.09) |
|  | **2013** | 0.9 (0.8;1.01) | 1.01 (0.88;1.15) |
| Month | **January** | Reference | Reference |
|  | **February** | 1.14 (0.96;1.34) | 1.23 (1.02;1.49)* |
|  | **March** | 1.03 (0.88;1.21) | 1.12 (0.93;1.34) |
|  | **April** | 0.98 (0.83;1.15) | 1.02 (0.85;1.22) |
|  | **May** | 1 (0.85;1.17) | 1.04 (0.87;1.24) |
|  | **June** | 0.94 (0.8;1.1) | 0.91 (0.76;1.09) |
|  | **July** | 0.93 (0.8;1.09) | 0.94 (0.79;1.12) |
|  | **August** | 0.94 (0.8;1.1) | 0.95 (0.8;1.14) |
|  | **September** | 0.92 (0.79;1.08) | 0.97 (0.81;1.16) |
|  | **October** | 1.08 (0.91;1.27) | 1.13 (0.94;1.36) |
|  | **November** | 0.92 (0.78;1.09) | 0.94 (0.78;1.13) |
|  | **December** | 1 (0.85;1.17) | 1 (0.83;1.2) |
| Day of week | **Sunday** | Reference | Reference |
|  | **Monday** | 1.05 (0.94;1.18) | 1.01 (0.89;1.15) |
|  | **Tuesday** | 1.03 (0.91;1.15) | 1.01 (0.89;1.16) |
|  | **Wednesday** | 1.05 (0.93;1.18) | 1 (0.88;1.14) |
|  | **Thursday** | 1.06 (0.94;1.19) | 1.02 (0.89;1.16) |
|  | **Friday** | 1.06 (0.95;1.2) | 1.04 (0.91;1.19) |
|  | **Saturday** | 0.99 (0.89;1.11) | 1 (0.88;1.13) |
| Time of Call | **0** | Reference | Reference |
|  | **1** | 1.09 (0.88;1.35) | 1.09 (0.86;1.38) |
|  | **2** | 1.15 (0.91;1.47) | 1.21 (0.93;1.58) |
|  | **3** | 1.45 (1.1;1.92)** | 1.88 (1.35;2.61)*** |
|  | **4** | 1.22 (0.92;1.62) | 1.29 (0.94;1.77) |
|  | **5** | 1.14 (0.84;1.55) | 1.04 (0.74;1.46) |
|  | **6** | 0.91 (0.67;1.23) | 0.92 (0.65;1.31) |
|  | **7** | 1.42 (1.03;1.96)* | 1.21 (0.84;1.74) |
|  | **8** | 2.29 (1.7;3.1)*** | 2.37 (1.68;3.34)*** |
|  | **9** | 1.46 (1.15;1.87)** | 1.52 (1.15;2)** |
|  | **10** | 1.56 (1.25;1.95)*** | 1.52 (1.18;1.96)** |
|  | **11** | 1.5 (1.22;1.84)*** | 1.49 (1.18;1.88)** |
|  | **12** | 1.46 (1.2;1.78)*** | 1.42 (1.14;1.78)** |
|  | **13** | 1.38 (1.14;1.67)** | 1.39 (1.12;1.73)** |
|  | **14** | 1.44 (1.2;1.74)*** | 1.35 (1.09;1.66)** |
|  | **15** | 1.33 (1.1;1.6)** | 1.37 (1.11;1.69)** |
|  | **16** | 1.35 (1.12;1.63)** | 1.33 (1.07;1.64)** |
|  | **17** | 1.35 (1.11;1.63)** | 1.32 (1.07;1.64)* |
|  | **18** | 1.34 (1.1;1.61)** | 1.3 (1.05;1.61)* |
|  | **19** | 1.36 (1.12;1.64)** | 1.3 (1.05;1.61)* |
|  | **20** | 1.26 (1.05;1.52)* | 1.25 (1.01;1.54)* |
|  | **21** | 1.14 (0.95;1.37) | 1.17 (0.95;1.44) |
|  | **22** | 1.2 (0.99;1.44) | 1.18 (0.96;1.46) |
|  | **23** | 1.1 (0.91;1.33) | 1.12 (0.91;1.38) |
| ICD10:X84 | **No** | Reference | Reference |
|  | **Yes** | 0.3 (0.28;0.33)*** | 1.23 (1;1.51)* |
| ICD9:950 | **No** | Reference | Reference |
|  | **Yes** | 6.61 (5.11;8.53)*** | 7.75 (5.83;10.31)*** |
| ICD9:980 | **No** | Reference | Reference |
|  | **Yes** | 2.63 (2.06;3.36)*** | 3.52 (2.59;4.77)*** |
| ICD9:305 | **No** | Reference | Reference |
|  | **Yes** | 2.43 (2.19;2.69)*** | 2.99 (2.44;3.67)*** |
| ICD9:969 | **No** | Reference | Reference |
|  | **Yes** | 6.9 (5.45;8.74)*** | 8.33 (6.38;10.86)*** |
| ICD9:300.9 | **No** | Reference | Reference |
|  | **Yes** | 6.19 (4.74;8.08)*** | 7.27 (5.48;9.64)*** |
| ICD9:V62.84 | **No** | Reference | Reference |
|  | **Yes** | 7.39 (2.32;23.52)** | 9.79 (3.05;31.46)*** |
| Priority | **Not Maximum** | Reference | Reference |
|  | **Maximum** | 2.93 (2.6;3.31)*** | 1.63 (1.4;1.9)*** |
